# D-Hexopyranosides with Vicinal Nitrogen-Containing Functionalities

**DOI:** 10.3390/molecules29153465

**Published:** 2024-07-24

**Authors:** Jana Pospíšilová, Daniel Toman, Tomáš Ručil, Petr Cankař

**Affiliations:** Department of Organic Chemistry, Faculty of Science, Palacký University Olomouc, 17. listopadu 1192/12, 77900 Olomouc, Czech Republic; jana.pospisilova10@upol.cz (J.P.); daniel.toman@upol.cz (D.T.); tomas.rucil@seznam.cz (T.R.)

**Keywords:** D-hexopyranosides, nitrogen-containing functionalities, glucosamine, aminoglycoside antibiotics, neuraminidase inhibitors, aziridine ring-opening, Michael addition, activated hydroxyl group

## Abstract

Various substituted D-hexopypyranosides units with nitrogen-containing functionalities are present in many important natural compounds and pharmaceutical substances. Since their complex structural diversity contributes to a broad spectrum of biological functions and activities, these derivatives are frequently studied. This review covers syntheses of D-hexopyranosides with vicinal nitrogen-containing functionalities since the 1960s, when the first articles emerged. The syntheses are arranged according to the positions of substitutions, to form a relative configuration of vicinal functionalities, and synthetic methodologies.

## 1. Introduction

The presence of nitrogen functionalities in saccharides increases their molecular complexity and diversity. The evolution of living organisms uses amino sugars for various functions, e.g., as structural components, signaling molecules, transporting molecules, and post-translational modified proteins.

The most commonly known *N*-acetylglucosamine is present in chitin as a monomer unit that forms the polysaccharide chain. Deacetylation of chitin leads to chitosan, which has practical applications in medicine, agriculture, and industry. Chitin is also processed to obtain D-glucosamine, which is frequently used as a dietary supplement and intermediate for biologically relevant molecules. Furthermore, the *N*-acetylglucosamine units are essential for the biosynthesis of peptidoglycans and hyaluronic acid.

Epimeric D-galactosamine units are present in glycoprotein hormones such as luteinizing and follicle-stimulating hormones [1]. D-Mannosamine, which is another epimer, has been mostly revealed in glycoproteins and gangliosides. The *N*-acetylated form is a precursor for the biosynthesis of *N*-acetylneuraminic acid, which is a predominant derivative of sialic acid in human cells [2]. *N*-Acetylneuraminic acid is also involved in the development of influenza virus infections and the biology of pathogenic and symbiotic bacteria [3,4].

The structure of *N*-acetylneuraminic acid was a starting point for the rational design of neuraminidase inhibitors. Thus, Zanamivir, which was the first commercially developed neuraminidase inhibitor, was approved for the treatment and prevention of influenza A and B. Oseltamivir, which is another commercial inhibitor with a more simplified structure, preserves two nitrogen functionalities. Recent ongoing development of these inhibitors relies on the bio-isosteric substitution to replace the carboxylate with a phosphonate or sulfonate group to increase the total binding energy [5].

The presence of nitrogen functional groups also plays an important role in the binding of aminoglycoside antibiotics such as Arbekacin, Kanamycin B, or Neomycin B, which bind to the bacterial ribosomal subunits. The amino saccharides were also used as starting materials to synthesize compounds with various biological activities. For example, the alkaloid (−)-Agelastatin A with anticancer activity and the glycophospholipid ligand of a lipopolysaccharide receptor were synthesized from D-glucosamine.

Nitrogen-containing functionalities in D-hexopyranosides have an irreplaceable role in living organisms. Thus, research teams continue to develop synthetic methodologies to introduce nitrogen-containing functionalities into D-hexopyranosides. This review focuses on syntheses that lead to D-hexopyranosides with vicinal nitrogen-containing functionalities.

**Figure 1:**
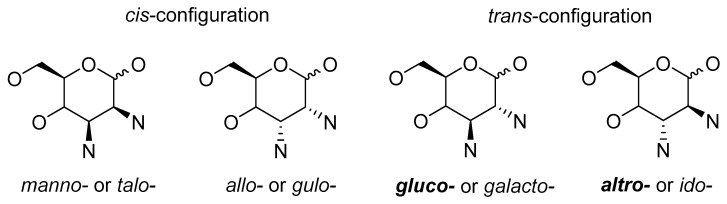
Configurations of 2,3–disubstituted D-hexopyranosides with vicinal nitrogen-containing functionalities.

Figure 1 defines the structures of interest of derivatives with nitrogen-containing functionalities at positions 2 and 3, and the special emphasis is on the most common molecules that contain *gluco*- and *altro*- configurations. These carbohydrates offer many biological activities and synthetic opportunities for further transformations. Several of them are discussed herein: (−)-agelastatin A, which has anti-tumor activity [6]; glycophospholipid PPDm2-B, which interacts with the liposaccharide receptor of macrophages [7]; ligands for the Mo-catalysed allylic alkylation [8]; half-sandwich metal complexes with comparable anti-tumor activity to *cis*-platina [9]; and hybrids of β-D-glucose with benzodiazepine scaffolds [10].

**Figure 2:**
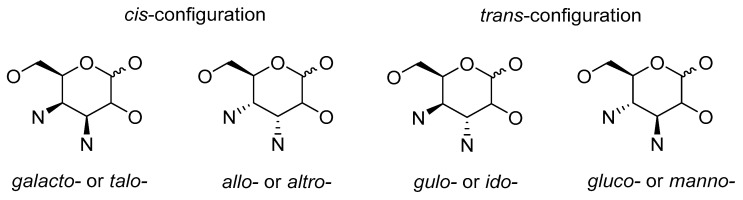
Configurations of 3,4-disubstituted D-hexopyranosides with vicinal nitrogen-containing functionalities.

Figure 2 shows the main motifs of the 3,4-disubstituted D-hexopyranosides. The *cis*-configuration of vicinal nitrogen-containing functionalities is in the derivatives of Neomycin, Kanamycin, and related compounds. The *trans*-configuration is incorporated in the skeleton of Zanamivir and its analogues.

## 2. Nitrogen Functionalities at Positions 2 and 3

Several synthetic methodologies provide D-hexopyranosides with nitrogen-containing functionalities at position 2 and 3. Generally, synthetic routes that lead to *cis*- and *trans*-derivatives are predetermined by the starting material. Both *trans*- and *cis*-derivatives are separately discussed.

### 2.1. Trans-Configuration

The derivatives with *trans*-oriented nitrogen-containing functionalities are synthesized based on the (a) aziridine formation and subsequent ring-opening reaction, (b) addition of an activated double bond, and (c) S_N_2 substitution of an activated hydroxyl group.

#### 2.1.1. Aziridine Formation

**Scheme 1:**
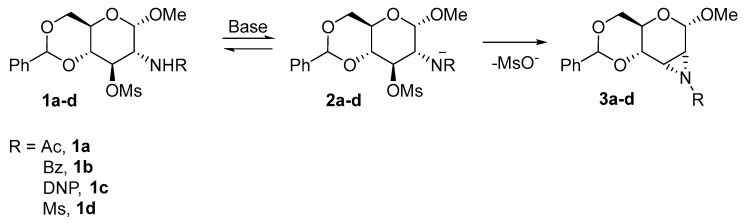
Formation of aziridine in the *allo*-configuration.

**Scheme 2:**
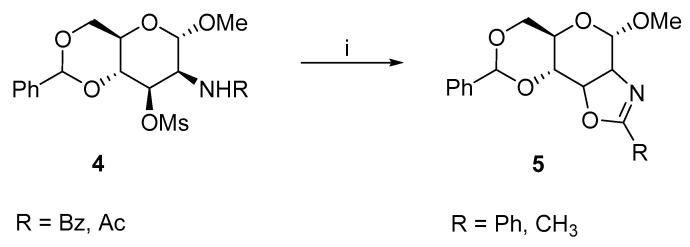
Formation of the oxazoline ring. Conditions: (i) EtONa, EtOH, reflux.

Richardson and coworkers described the formation of aziridine **3** with an *allo*-configuration from substituted glucopyranosides **1a–d** in 1965 (Scheme 1). The crucial condition for aziridine formation is the *trans-diaxial* configuration of glucosamine **1**. The *cis*-configuration (e.g., in mannopyranoside) results in hexopyranosides **5** with an oxazoline ring (Scheme 2) [11]. A base-catalyzed aziridine formation requires strong nucleophilic anion of the acylamido group at the C2 carbon, which attacks the C3 carbon with the activated hydroxyl group to yield the desired aziridines **3a–d**. There are many suitable bases; the method of choice predominantly depends on the *N*-substitution.

**Scheme 3:**
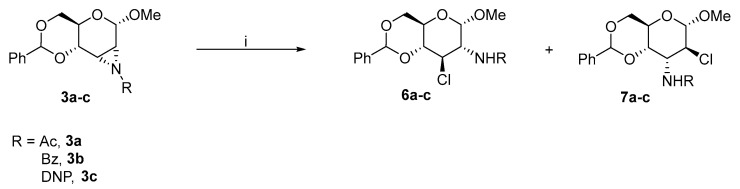
Opening of *allo*-aziridine with ammonium chloride. Conditions: (i) NH_4_Cl, DMF, reflux.

Richardson inspired other research groups. Later, Richardson [12] extended the substitution at C2–amine to anisoyl, dinitrophenyl (DNP), and mesyl groups with the desired activating effect and described the possibilities for ring-opening reactions. The product of the ring-opening reaction strongly depends on the amine substitution at the C2 carbon and nucleophile. As mentioned (in Scheme 1), a stronger anion favours the aziridine formation. A weaker anion could not accomplish the substitution of the *O*-mesyl group, and oxazoline **5** was obtained as a major side product. The same publication described the ring-opening reactions of acetylated, benzoylated, or DNP-substituted aziridines **3a–c** with ammonium chloride. Initially, the ring-opening reactions were accomplished with halogen nucleophiles. When aziridines **3a–c** were refluxed in DMF, the aziridine underwent *trans-di-axial* and *trans-di-equatorial* ring-opening reactions and formed *gluco*- (**6a–c**) and *altro*- (**7a–c**) products (Scheme 3).

**Scheme 4:**
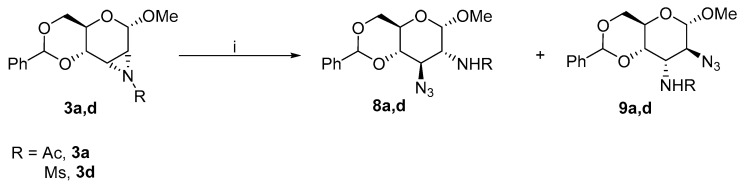
Ring-opening reaction of *allo*-aziridine with sodium azide in the presence of ammonium chloride. Conditions: (i) NaN_3_, NH_4_Cl, DMF, and reflux.

The treatment of **3a** with ammonium chloride provided almost exclusively *gluco*- derivative **6a**, whereas aziridines **3b** and **3c** yielded mixtures of **6b**,**c** and **7b**,**c**, respectively. However, when sodium azide, which is a stronger nucleophile, was added to a reaction mixture dissolved in DMF in the presence of ammonium chloride, the formation of chloro- derivatives was suppressed, and azido derivatives **8** and **9** were formed (Scheme 4).

**Scheme 5:**
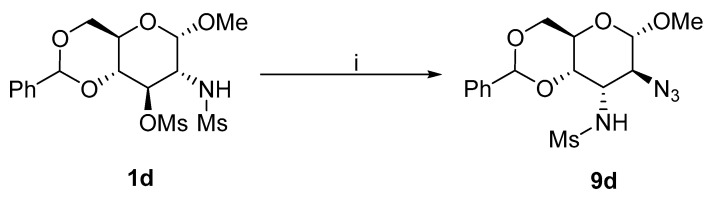
Ring-opening reaction of in situ formed aziridine with sodium azide. Conditions: (i) NaN_3_, DMF, reflux, 65%.

Aziridines **3a** and **3d** provided unsatisfactory yields of products with a mixture of ammonium chloride and sodium azide. The formation of side products or degradation of a starting compound was observed. Therefore, **1d** was reacted with sodium azide in the absence of ammonium chloride to yield more **9d** (Scheme 5).

**Scheme 6:**
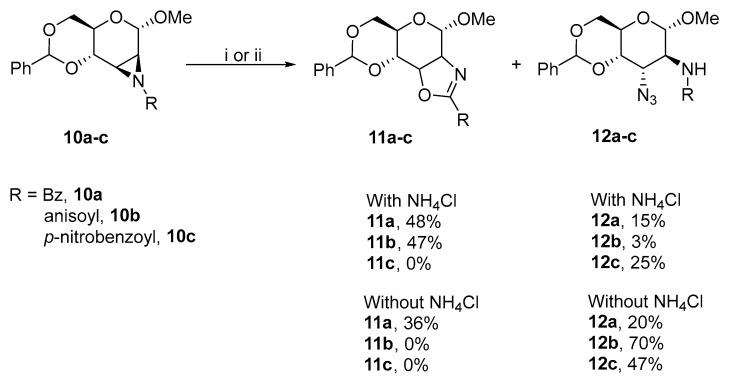
Ring-opening reaction of *manno*-aziridine with sodium azide in the presence or absence of ammonium chloride. Conditions: (i) NaN_3_, DMF, and reflux; (ii) NaN_3_, NH_4_Cl, DMF, and reflux.

The reaction proceeded via in situ formation of aziridine and a *trans-diaxial* ring-opening reaction, which exclusively resulted in **9d** with the *altro*-configuration. With Richardson and coworkers, the Guthrie group [13,14] investigated the ring-opening reactions of *manno*-aziridine (Scheme 6). The reaction of **10b** with sodium azide provided the highest yields. A similar conclusion was reached also by Meyer zu Reckendorf [15,16,17], who described the formation of an additional *gluco*-derivative.

**Scheme 7:**
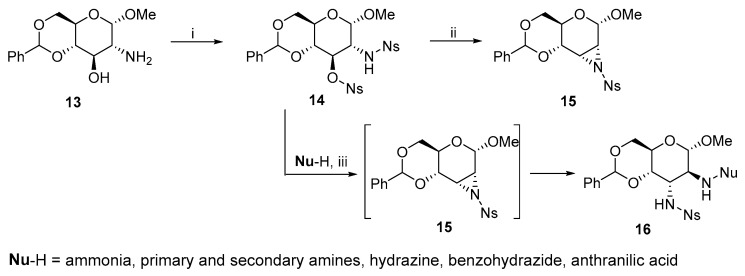
Ring-opening reactions of *N*-4-nosyl Hough–Richardson aziridine. Conditions: (i) 4-NsCl, DCM/Py, 60 °C, 72 h, 70%; (ii) DIPEA, 2-methoxyethanol, 60 °C, 24 h, 83%; and (iii) DIPEA, DMSO, 60 °C, 16–72 h, 38–91%.

The scope of the substitution at positions 2 and 3 was later expanded by ring-opening reactions of *N*-4-nosyl Hough–Richardson aziridine with 19 nitrogen nucleophiles (Scheme 7) [18]. The electron-withdrawing nitro group provided practical advantages. Aziridine **15** was synthesized under mild conditions with high yields. This aziridine can also be generated without isolation to furnish product **16** with an *altro*-configuration due to the highly regioselective ring-opening reactions. The *altro*-configuration is preferred over the *gluco*-configuration at ratios above 90:10.

**Scheme 8:**
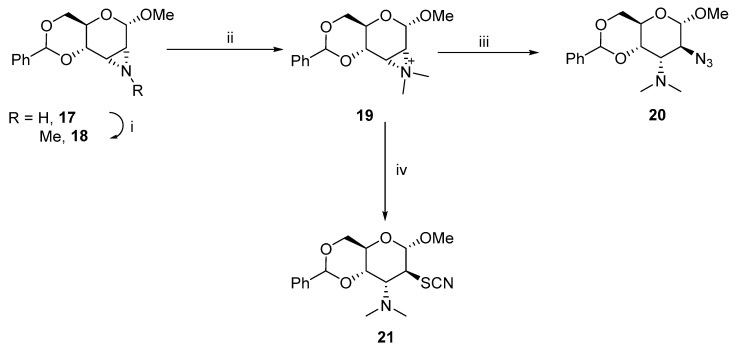
Ring-opening reaction of *allo*-aziridinium salt with sodium azide and potassium thiocyanate. Conditions: (i) MeI, Ag_2_CO_3_, MeOH, 62%; (ii) MeI, silver pycrilsulphonate, CAN, 66% (iii) NaN_3_, DMF, 22%; and (iv) KSCN, DMF, 57%.

D-hexopyranosides with vicinal *trans*-oriented nitrogen-containing functionalities can also be synthesized via aziridinium salts (Scheme 8) [19]. The hydrolysis of **19** with sodium azide and potassium thiocyanate yielded **20** and **21**, which exclusively had *altro*-configurations.

**Scheme 9:**
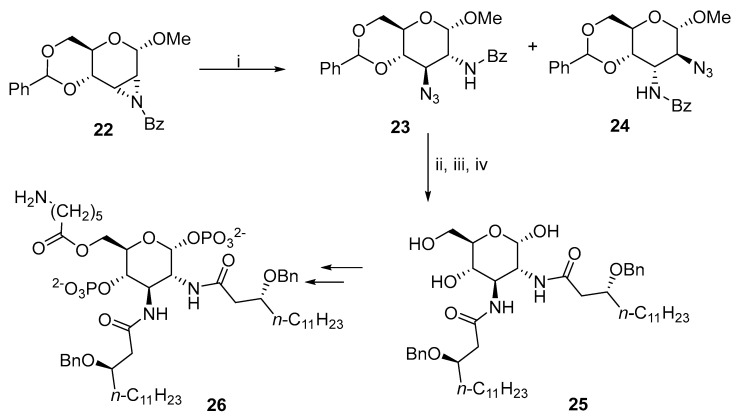
Synthesis of glycophospholipid ligands **26**. Conditions: (i) NaN_3_, NH_4_Cl, DMF, 24 °C, 25% for **23**, 70% for **24**; (ii) H_2_, Pd(OH)_2_/C, MeOH, (iii) 5 M HCl, 100 °C, 68% (three steps); and (iv) *(D)*-3-OBn-Myr-O-C_4_H_2_NO_2_, *i*-Pr_2_NEt, DMF, 43%.

The works of Richardson, Guthrie, and Meyer zu Reckendorf laid the foundations for further applications in the ring-opening reactions. Charon and co-workers used an aziridine ring-opening reaction to synthesize glycophospholipid ligands of lipopolysaccharide receptor **26** (Scheme 9) [7]. The ring-opening reaction of **22** afforded *gluco*-**23** as a minor and *altro*-**24** as a major product.

**Scheme 10:**
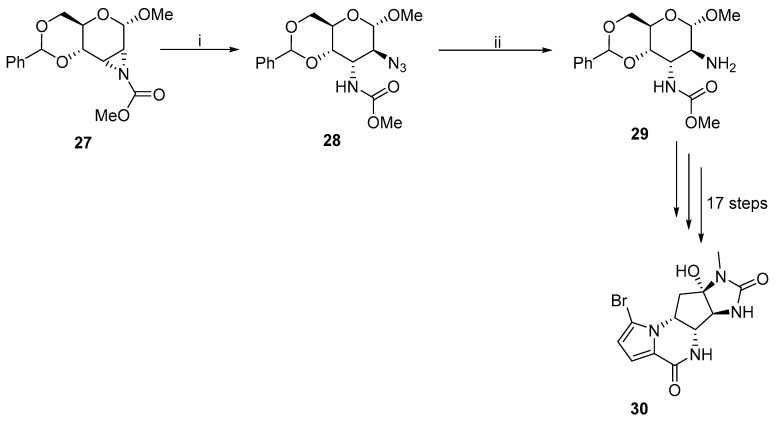
Ring-opening reaction to synthesize Agelastatin A. Conditions: (i) NaN_3_, NH_4_Cl, DMF, 88%; (ii) H_2_, Pd(OH)_2_, and MeOH, 95%.

Hale used the aziridine-opening reaction to synthesize Agelastatin A **30**, which is an inhibitor of GSK-3*ß* (Scheme 10) [6]. Aziridine **27** was treated with sodium azide in the presence of ammonium chloride, and **28** was a major product. No minor product was separated. Azide **28** was subsequently reduced to give derivative **29** at a very good yield. The desired Agelastatin A **30** was synthesized in a 17-step process.

#### 2.1.2. Substitution of the Activated Hydroxyl Group

**Scheme 11:**
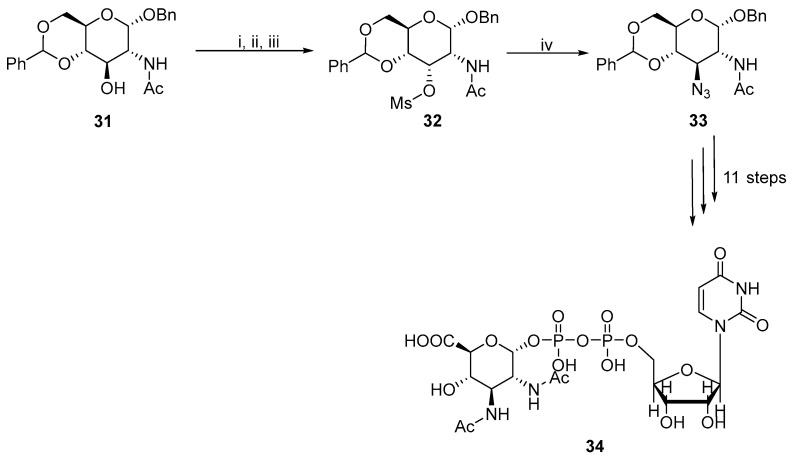
C3 double inversion to synthesize a derivative of glucuronic acid. Conditions: (i) MsCl, pyridine; (ii) NaOAc, 2-methoxyethanol, H_2_O; (iii) MsCl, pyridine; and (iv) NaN_3_, TBAHS, DMF, 73% (overall yield).

Nucleophilic substitution, which is associated with the inversion of a configuration, was performed after the hydroxyl group with mesyl, tosyl, or triflate agents had been activated. Rejzek and coworkers prepared phospho-derived glucuronic acid **34** using a double inversion at the C3 carbon (Scheme 11) [20].

**Scheme 12:**
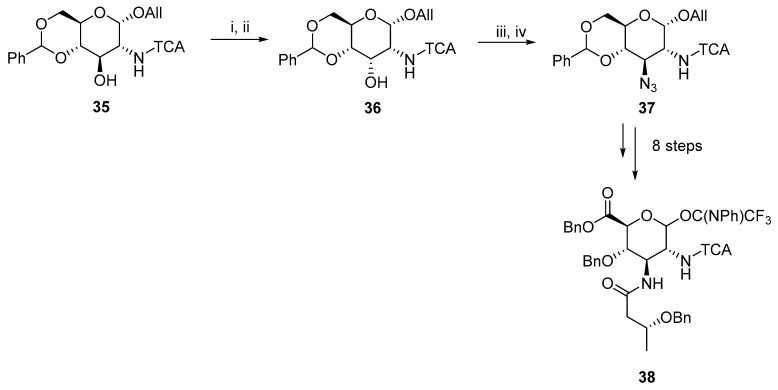
Synthesis of 2,3-diamino-D-glucuronate **38**. Conditions: (i) Tf_2_O, Py, DCM, −20 °C; (ii) KNO_2_, DMF, 50 °C, 6 h, 71% (two steps); (iii) Tf_2_O, Py, DCM, −20 °C, 4 h; and (iv) NaN_3_, DMF, rt, 59% (two steps).

A new strategy to synthesize 2,3-diamino-D-glucuronate was published for the total synthesis of *Plesiomonas shigelloides* serotype 51 aminoglycoside trisaccharide [21]. An interesting part of this synthesis was the Lattrel-Dax inversion from *gluco*- to *allo*-derivative **36**. The described double inversion at C3 was first used to assemble a complex aminoglycoside **38** with various substitutions (Scheme 12). A trichloroacetamido (TCA) group was selected to mask the acetamido group of **38** to stereoselectively form the β-glycosidic bond.

**Scheme 13:**
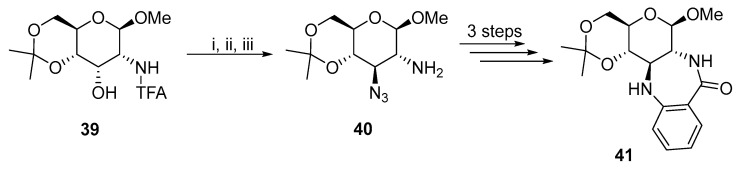
Formation of the benzodiazepinone scaffold. Conditions: (i) MsCl, TEA, DCM, 91%; (ii) NaN_3_, Bu_4_NHSO_4_, DMF, 100 °C, 92%; and (iii) 5 M KOH, MeOH, reflux, 99%.

A similar approach was used to synthesize cinnamon derivatives [22], 2,3-*trans*–diamino–metal-complexes (Mn, Pt, Rh, Ru, Ir, Cu, and Pd) [23,24,25,26,27,28,29,30,31], muramyl dipeptide analogues [32], or ß-D-glucose benzodiazepine derivatives **41 [10]** (Scheme 13).

#### 2.1.3. Michael Addition: Using Addition to Activated Double Bond

**Scheme 14:**
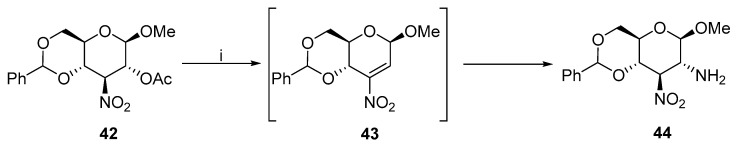
Michael addition of ammonia to 3-nitroolefine. Conditions: (i) Ammonia, dry THF, 86%.

Michael addition to nitroolefins is another method to obtain 2,3–*trans*-diaminohexopyranosides. Baer reported the formation of olefins followed by the addition of ammonia in THF (Scheme 14) [33]. In addition to **44**, an impurity with a yield below 10% was isolated but not characterized.

**Scheme 15:**
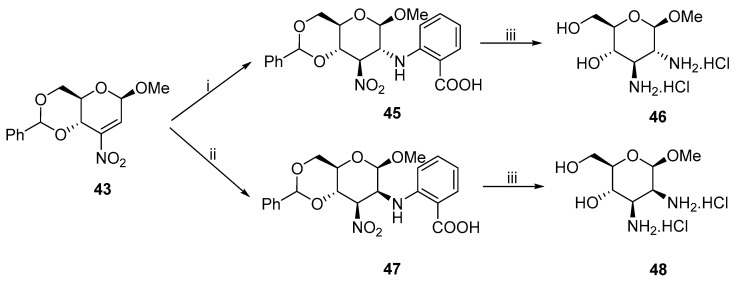
Addition of anthranilic acid to 3-nitroolefine. Conditions: (i) anthranilic acid, KOH, dry benzene, 83%; (ii) anthranilic acid, dry benzene, 56%; and (iii) H_2_, Pd, BaSO_4_, 1 M HCl, 60–70%.

Afterwards, the synthesis was extended to anthranilic acid [34] and aminosugars [35]. The configuration of the diamine strongly depends on the pH of the reaction (Scheme 15). When the reaction occurred under basic conditions, *gluco*-product **45**, which is thermodynamically more stable, was almost exclusively formed. In contrast, without KOH, *manno*- product **47** was isolated as a major isomer. Both **45** and **47** underwent reduction in the presence of the Kuhn catalyst and yielded diamines **46** and **48**.

**Scheme 16:**
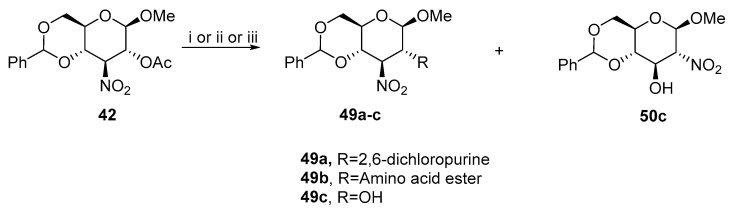
Addition of various nitrogen nucleophiles to 3-nitro derivative **42**. Conditions: (i) 2,6-dichloropurine, Na_2_CO_3_, THF, 78%; (ii) amino acid ester, Et_3_N, THF/MeOH, 73–88%; and (iii) NaNO_2_, Amberlite IRC 50, CAN/water, 70%.

Subsequent publications used nucleosides [36], esters of amino acids [37], or sodium nitrite [38]. The reaction with 2,6-dichloropurine and amino acids esters (Gly, Ala, Phe, Ser, Tyr, and Val) exclusively produced **49a** (a C–N bond was formed between C2 carbon and nitrogen at position 9) and **49b**, respectively (Scheme 16). However, treating **42** with sodium nitrite resulted in 3-nitro derivative **49c** as a major product and **50c** as a minor isomer. The ratio of **49c** to **50c** could be slightly increased by adding hexadecyltributylphosphonium bromide.

**Scheme 17:**
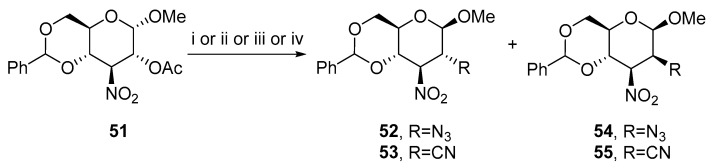
Study of the substitution with azide and cyanide nucleophiles. Conditions: (i) NaN_3_ ACN/water, 60% (for **52**); (ii) HN_3_, THF/water, 79% (for **54**); (iii) HCN, KCN, ACN, 42% (for **54**); and (iv) HCN, DMSO, yield not specified.

Sakakibara and Sudoh studied the influence of the solvent and reagent on the substitution with azide or cyanide nucleophiles (Scheme 17) [39]. When sodium azide was used, **52** was isolated in 60% yield. When hydrazoic acid was added to THF, epimeric **54** was obtained in 79% yield. Therefore, more solvents were examined. Solvents such as DMSO or THF in the presence of hydrazoic acid favor the formation of **54**. Chloroform or acetonitrile produced a mixture of **52** and **54**. The study with hydrogen cyanide and potassium cyanide obtained the same conclusion. The reaction in DMSO led to major product **55** with the *manno*- configuration and the substitution in acetonitrile provided a mixture of **53** and **55**.

**Scheme 18:**
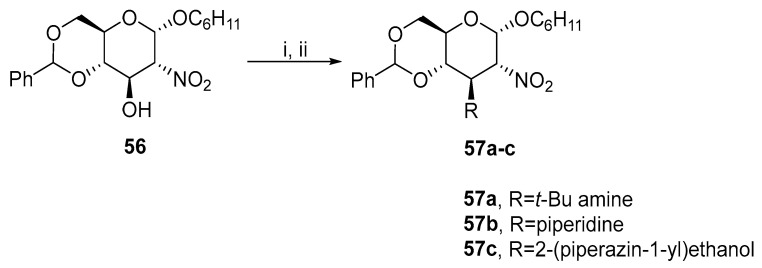
Addition of primary or secondary amines to in situ generated 2-nitroolefin. Conditions: (i) MsCl, Et_3_N, DCM; and (ii) amine, DCM, (two steps, 88–92%).

The nitro group was also used at the C2 carbon (Scheme 18). Starting compound **56** was readily obtained by oxidizing protected glucosamine with *m*-CPBA [40]. Product **57c** was further tested as a drug carrier.

### 2.2. Cis-Configuration

The cis configuration is commonly introduced by substituting an activated hydroxyl group with the appropriate configuration. Other less frequently used synthetic methods include subchapter miscellaneous reactions.

#### 2.2.1. Substitution of Activated Hydroxyl Group

**Scheme 19:**
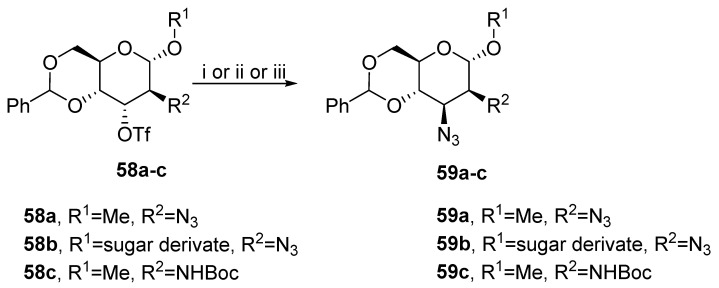
Nucleophilic substitution of the activated hydroxyl group to yield mannoside derivatives. Conditions: (i) NaN_3_, NH_4_Cl, DMF, 75%; (ii) tetramethylguanidinium azide, DCM, 68%; and (iii) *n*-Bu_4_NN_3_, pyridine/DCM, 70%.

The most common method to prepare vicinal *cis*-oriented nitrogen-containing derivatives is based on nucleophilic substitution. Due to the inevitable inversion of the configuration during substitution, the hydroxyl group in the starting compound must be in the *trans*- position to the amine functional group. Walvoort used altropyranoside (**58a**) to synthesize mannopyranoside uronates. The number of azide groups was reduced in **59a**, and the resulting intermediate was transformed into more complex derivatives (Scheme 19) [41]. Baer prepared disaccharose of the trehalose type **59b** [42]. Finally, **59c** was synthetized as a substrate for *N*-acetylneuraminic acid aldolase [43].

**Scheme 20:**
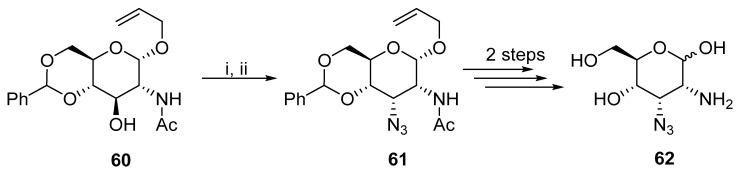
Synthesis of catalytic cofactor analogues for glmS Rybozime. Conditions: (i) Ms_2_O, pyridine; (ii) NaN_3_, DMF, 52% (two steps).

In contrast, Posakony prepared allopyranoside carbohydrate **61** from **60**, where the final product **62** could serve as a catalytic cofactor analogue for glmS Rybozime (Scheme 20) [44]. The desired change in the configuration was achieved by the reaction of sodium azide with the mesylated hydroxyl group.

**Scheme 21:**
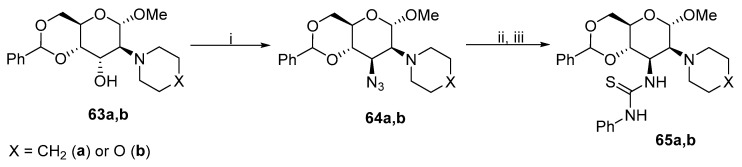
Mitsunobu reaction to synthesize new organocatalysts. Conditions: (i) DPPA, TPP, DIAD, THF, 60–80%; (ii) TPP, THF/water; and (iii) Phenylisothiocyanate, MeOH, 80% (two steps).

Alternatively, Mitsunobu reaction was used instead of the classical nucleophilic substitution of the activated hydroxyl group. This synthetic tool was applied to synthesize carbohydrate-based organocatalysts, where the C3 hydroxyl group was azidated with DPPA under Mitsunobu conditions (Scheme 21) [45].

#### 2.2.2. Miscellaneous Methods

**Scheme 22:**
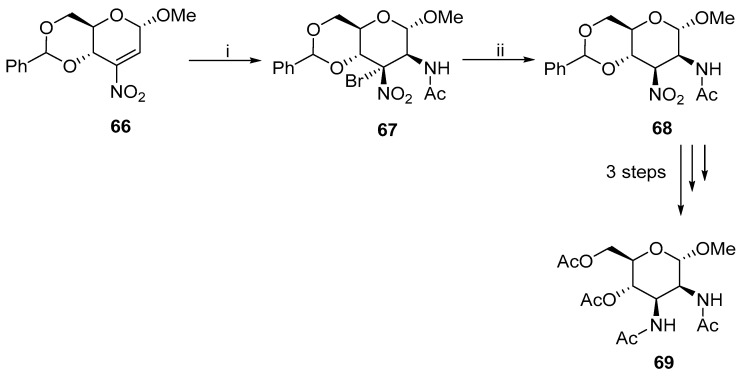
Addition of *N*-bromoacetamide. Conditions: (i) *N*-bromoacetamide, NaOAc, acetone, 76%; (ii) NaBH_4_, EtOH, 91%.

In addition to the S_N_2 reactions, the *cis*-configuration was achieved by other reactions. For example, Rank synthesized **69**, where the key step was the addition of *N*-bromoacetamide to 3-nitroolefin **66** (Scheme 22) [46]. The reduction of **67** afforded nitro derivative **68**, which was converted to acetylated mannopyranoside **69** in a three-step process. Similar results were obtained when talopyranoside was used.

**Scheme 23:**
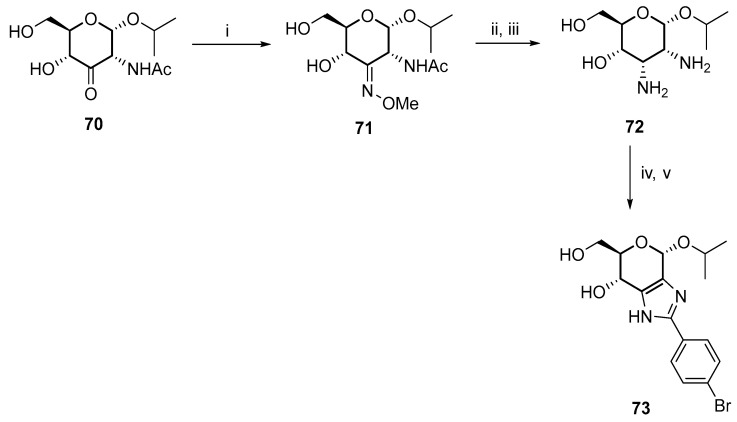
Synthesis of diaminoallose and the corresponding imidazole-fused derivative. Conditions: (i) MeONH_2_.HCl, NaHCO_3_, MeOH, 96%; (ii) H_2_, PtO_2_, AcOH, 80%; (iii) 1 M NaOH, MW 150 °C, 85%; (iv) 4-bromobenzaldehyde, *N*-iodosuccinimide, *t*-BuOH, 51%; and (v) PdI(OAc)_2_, K_2_CO_3_, DMSO, 42%.

Reductive amination at the C3 carbonyl provided another effective route to 3-aminoglucose (Scheme 23) [47]. Methyl oxime was formed as a mixture of the *E* and *Z* isomers, and subsequent hydrogenation provided the *axially*-oriented 3-amino group due to the anomeric isopropyl substituent. Furthermore, 2,3-dideoxy-2,3-diaminoallose **72** was used as a building block to synthesize imidazole derivative **73**.

**Scheme 24:**
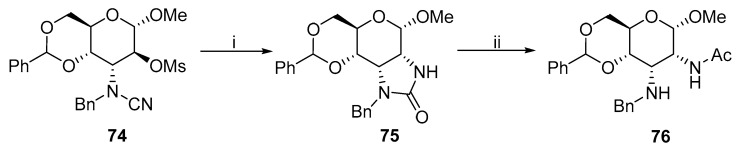
Synthesis of allopyranoside. Conditions: (i) NH_3_/EtOH, 72%; (ii) KOH, glacial CH_3_COOH, Ac_2_O, 34%.

Another alternative method includes the formation of an imidazoline ring and its subsequent basic hydrolysis (Scheme 24). Baker et al. prepared 2,3–diamino *allo*-pyranosides **76** from the corresponding imidazolines **75**, where phenyl could be attached to the imidazoline nitrogen instead of benzyl [48,49,50,51]. The long reaction time (up to 5 days) in each step was the main disadvantage of these synthetic routes.

**Figure 3:**
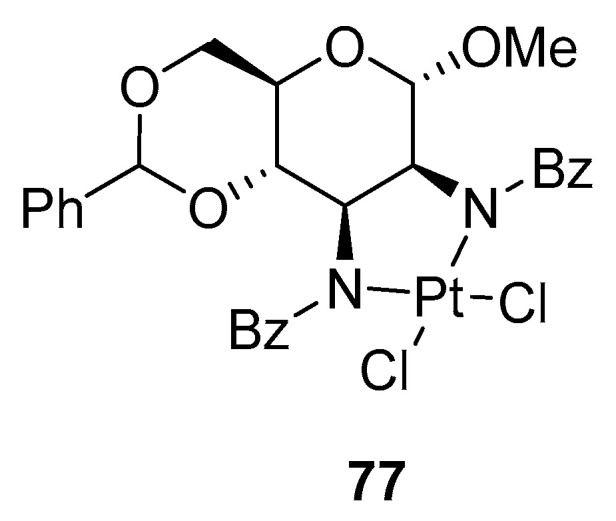
Structure of the platinum complex.

Then, Baker et al. suggested the use of tosyl instead of benzyl groups, but the long reaction time and low yields unfortunately remained. Recently, *allo*- or *manno*-pyranosides were applied in the chemistry of complexes, where carbohydrates were used as ligands **77** and exhibited comparable anti-tumor activity comparable to *cis*-platina (Figure 3) [29].

## 3. Nitrogen Functionalities at Positions 3 and 4

The first part focuses on the synthesis of *trans*-diaminohexopyranoses, which are mainly incorporated into the skeleton of Zanamivir and its analogues. The second section discusses *cis*-dinitrogen-containing D-hexopyranoses, particularly derivatives of Neomycin, Kanamycin and related compounds. The final part discusses the reactions that lead to *cis*- and *trans*- products, where the configuration depends on the reaction conditions.

### 3.1. Trans-Configuration

**Figure 4:**
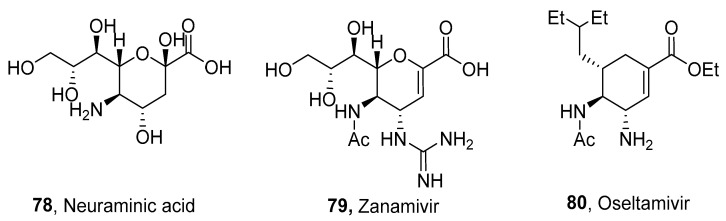
Structure of neuraminic acid **78** and neuraminidase inhibitors.

A *trans* configuration with nitrogen-containing functionalities at positions 3 and 4 was introduced, particularly in the compounds derived from Neuraminic acid **78** (Figure 4); however, the *trans* configuration was found in other structures. Some neuraminidase inhibitors, such as Zanamivir **79** or Oseltamivir **80** are commercially available. These derivatives exhibit antiviral properties; therefore, their substitution is a topic of many research studies. There are several synthetic approaches to obtain the *trans* configuration: (a) oxazoline ring formation, (b) cyclization of acyclic intermediates with vicinal dinitrogen-containing functionalities, (c) Michael addition, and (d) aziridine ring formation.

#### 3.1.1. Oxazoline Ring Formation

**Scheme 25:**
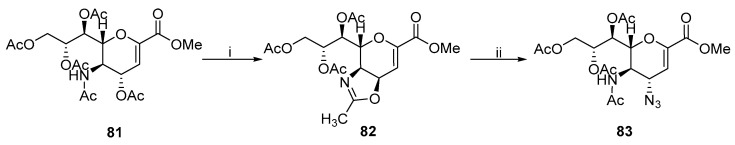
Formation and ring-opening reaction of the oxazoline ring. Conditions: (i) BF_3_.Et_2_O, DCM, MeOH, 99%; and (ii) LiN_3_, Dowex 50W, DMF, 96%.

The formation and ring-opening of the oxazoline ring constitute a proven route to synthesize *trans*-3,4–diamino carbohydrates. Von Itzstein et al. prepared oxazoline **82** from *O*-acetylated derivative **81** (Scheme 25) [52]. The oxazoline ring is vulnerable to nucleophilic attacks at the C-O bond. Treating **82** with lithium azide forms **83**.

**Figure 5:**
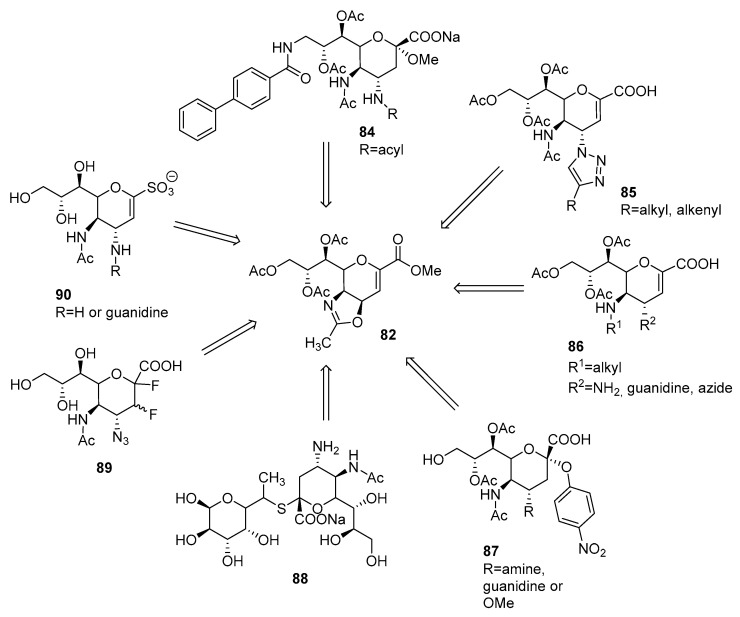
Synthesis of Zanamivir derivatives by forming an oxazoline ring and modifications.

The ring-opening reaction of the oxazoline ring with an azidation reagent was used to synthesize many derivatives. The substitution of deprotected primary hydroxyl group and a subsequent oxazoline ring-opening reaction with TMSN_3_ and azide reduction afforded carbohydrate **84** (Figure 5) [53]. The click reaction of azide with various acetylenes yielded triazole derivatives **85** [54]. Moreover, azide was reduced and converted to guanidine **86** [54,55,56,57]. The substitution at the anomeric hydroxyl group afforded product **87** [56]. The second sugar unit could be connected with the thioether bond and produced **88** [58]. A protocol to synthesize fluoro diastereomers **89** was described [59]. The reduction of the azide moiety of **83** and further insertion of the sulfonic acid group at position 1 yielded sialosyl α-sulfonate derivatives **90**, which significantly inhibited the influenza virus sialidase activity [60].

**Scheme 26:**
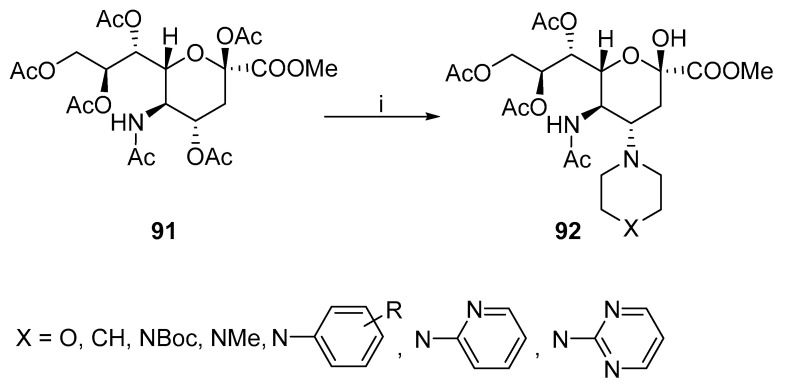
Substitution of morpholine via an oxazoline intermediate at position 4. Conditions: (i) pyridine, morpholine, piperidine, or derivatives of *N*-substituted piperazine, and room temperature.

In addition to the most commonly used azide reagents, the oxazoline ring can be opened by other nucleophiles. Ye et al. published a protocol to introduce morpholine, piperazine, piperidine, pyrrolidine, or primary and secondary amino moieties to obtain derivative **92** (Scheme 26) [61,62,63]. The protocol included an in situ formation of an oxazoline ring, which immediately proceeded to nucleophilically attack morpholine. The acetoxy group at position 6 also participates in the ring-opening reaction and undergoes selective deacetylation. This methodology was expanded by Bozzola et al. with various substituted *N*-aryl and *N*-heteroaryl piperazine derivatives [64].

**Figure 6:**
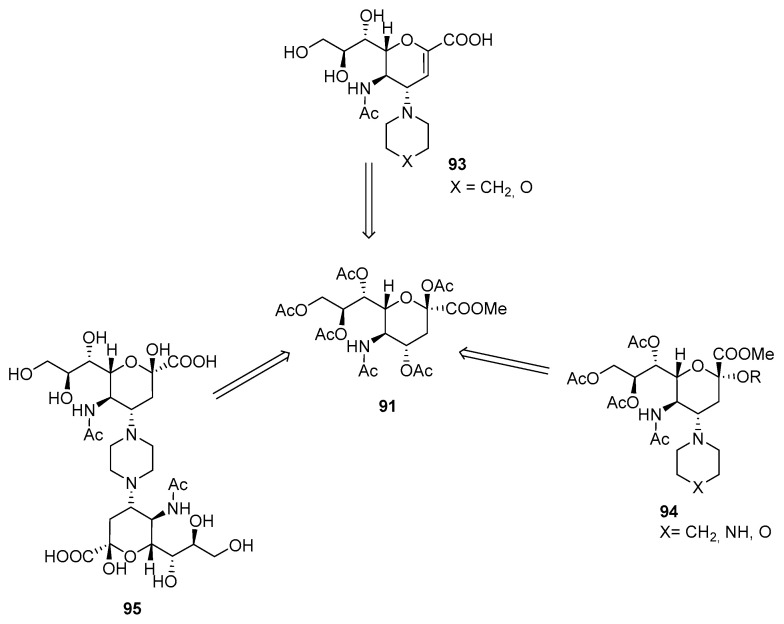
Synthesis of various derivatives via an oxazoline ring-opening reaction.

This method was later used to synthesize other Zanamivir derivatives. Rota et al. (Figure 6) prepared Zanamivir derivatives **93** in a four-step synthesis [65], Further substitution was performed at the anomeric carbon of **94** [66], where various alcohols were used as nucleophiles. Then, **95** was synthesized via double ring-opening reactions to connect two carbohydrate units through the piperazine linker [63]. The linker can be extended with further substitution at piperazine.

#### 3.1.2. Cyclization of Acyclic Intermediates with Vicinal Dinitrogen-Containing Functionalities

**Scheme 27:**
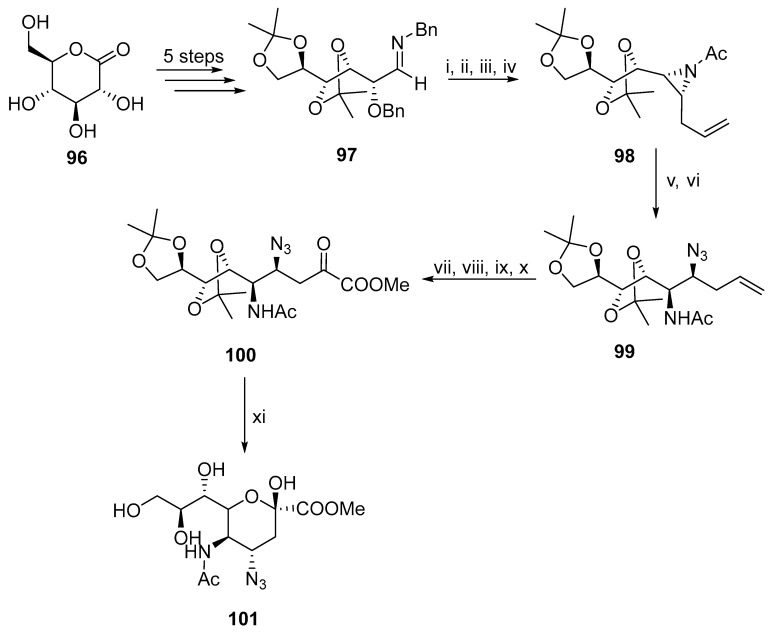
Synthesis of reactive intermediate **100**. Conditions: (i) C_3_H_5_MgBr, Et_2_O, 56%; (ii) Ac_2_O, Et_3_N, DCM, 88%; (iii) Li, NH_3_, THF, 82%; (iv) NaH, THF, 87%; (v) NaN_3_, NH_4_Cl, EtOH/water, 62%; (vi) Ac_2_O, Et_3_N, DMAP, DCM, 88%; (vii) OsO_4_, NMO, acetone/water, 96%; (viii) KBr, TEMPO, TBAB, Ca(ClO)_2_, DMF; (ix) MeI, K_2_CO_3_, DMF, 80%, (2 steps); (x) DMP, DCM; and (xi) 40%HF in MeCN 52% (two steps).

An alternative to the oxazoline ring-opening reaction is the cyclization of **100** (Scheme 27). The reaction sequence begins with lactone **96**, where a five-step synthesis results in imine **97** [67]. Then, imine **97** is converted to aziridine **98** in a four-step synthesis [68]. Intermediate **99** is synthesized through an aziridine ring-opening reaction. The acidic cyclization of ketoester **100** yields **101**.

**Scheme 28:**
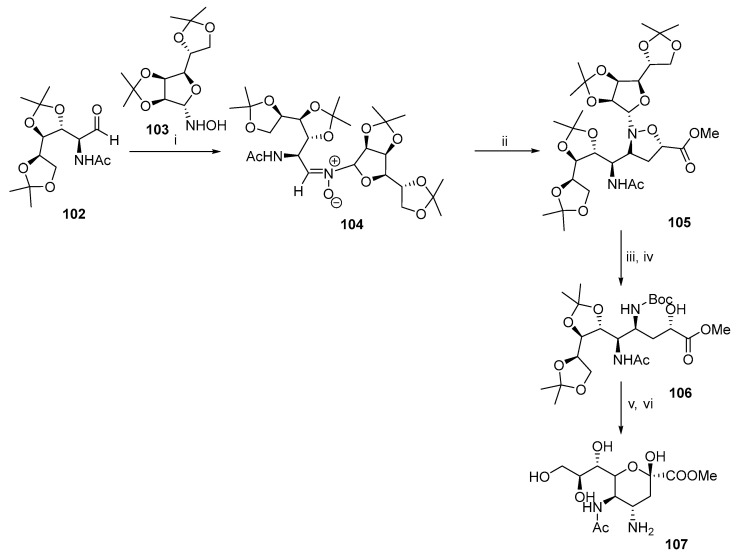
Alternative synthesis of **107** via an isoxazolidine intermediate. Conditions: (i) MgSO_4_, DCM, 53%; (ii) methyl acrylate, toluene, 90%; (iii) NH_2_OH, NaOAc, MeOH/water, 59%; (iv) Pd(OH)_2_, then Boc_2_O, 100%, (v) DMP, DCM; and (vi) 4 M HCl, THF, then Et_3_N, 70% (two steps).

Yao and co-workers developed an alternative synthesis of the similar derivative **107**. Air-stabilized nitrone **104** was prepared by adding hydroxylamine derivative **103** to aldehyde **102** (Scheme 28) [69,70]. Heating **104** with methyl acrylate yielded isoxazolidine **105**, which was hydrolyzed, and the N-O bond was subsequently cleaved to obtain alcohol **106**. Dess-Martin oxidation afforded the keto intermediate, which was cyclized under acidic conditions to obtain **107**.

**Scheme 29:**
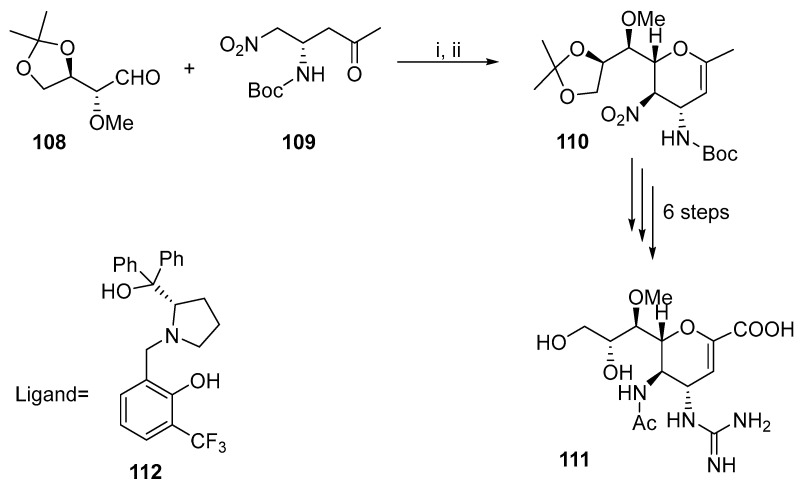
Synthesis of Zanamivir derivative **111** from key intermediate **110**. Conditions: (i) CuBr_2_, ligand, Cs_2_CO_3_, THF, 0 °C; and (ii) SOCl_2_, pyridine/DCM, 0 °C, 76% (two steps).

Another method to construct a reactive intermediate suitable for ring closure is the Henry reaction (Scheme 29) [71]. An *anti*-selective Henry reaction in the presence of ligand **112** yielded the cyclic intermediate, which is dehydrated by thionyl chloride and pyridine to produce **110**. Nitro derivative **110** was used as the starting material for the six-step synthesis of Zanamivir derivative **111**. This synthesis can be conducted on a large scale.

**Scheme 30:**
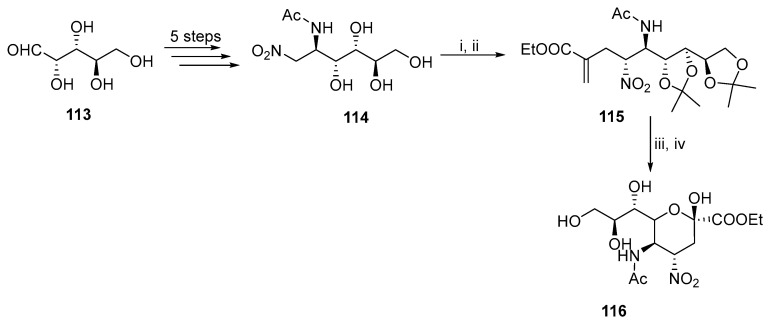
Cyclization resulting in **116** after ozonolysis and deprotection. Conditions: (i) acetone, CuSO_4_, H_2_SO_4_, 74%; (ii) ethyl α-(bromomethyl)acrylate, NaOH, water, 81%; (iii) O_3_, MeOH/DCM; and (iv) dimethylsulfide, 55% (two steps).

The final example of cyclization is based on the key alkylation step (Scheme 30) [72]. Mannitol derivative **114** was prepared from arabinose **113** in a five-step synthesis. Subsequent protection and alkylation with ethyl α-(bromomethyl)acrylate formed acyclic intermediate **115**, which produced **116** after ozonolysis and subsequent reductive deprotection.

#### 3.1.3. Michael Addition

**Scheme 31:**
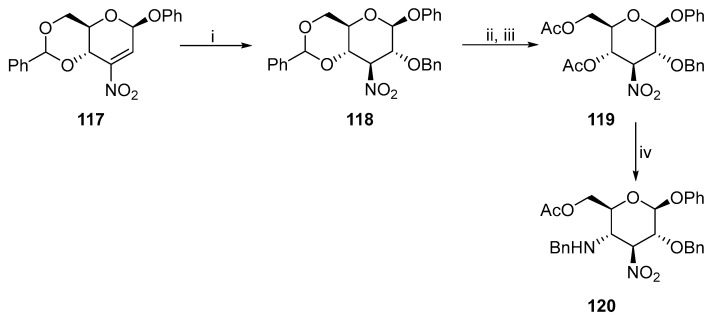
Substitution of –OAc with a primary amine. Conditions: (i) BnOH, Et_3_N, toluene, 64%; (ii) 90% AcOH, 60%; (iii) Ac_2_O, pyridine, 72%; and (iv) BnNH_2_, THF, 77%.

A primary amine was introduced by Michael addition to a D-glucosamine derivative [73]. The key step to synthesize **120** (Scheme 31) involves eliminating acetic acid and subsequent Michael addition of benzylamine.

#### 3.1.4. Aziridine Ring Formation

**Scheme 32:**
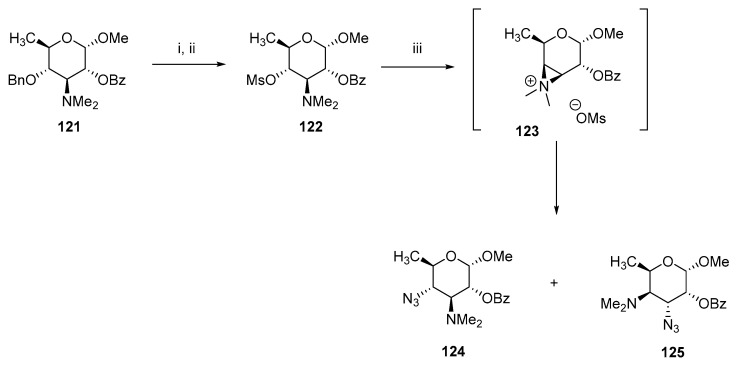
Synthesis of two azido-modified desosamine derivatives **124** and **125**. Conditions: (i) Pd(OH)_2_/C, H_2_, 1 atm, AcOH (1%), MeOH, 82%; (ii) MsCl, Py, RT, 90%; and (iii) NaN_3_, DMF, 100 °C, 52% for **124**, 31% for **125**.

In several cases, *trans* 3,4-diaminocarbohydrates were synthesized through the aziridine salt intermediate. Chen et al. prepared a mixture of azido modified desosamines **124** and **125** (Scheme 32) [74]. The synthesis began with 4-*O*-benzyl mycaminose **121**, which was converted into reactive 4-mesyl intermediate **122** in two steps. Treatment of **122** with NaN_3_ produced two isomers **124** and **125**, which indicates the in situ formation of aziridinium intermediate **123** and inevitable nucleophilic ring opening.

**Scheme 33:**
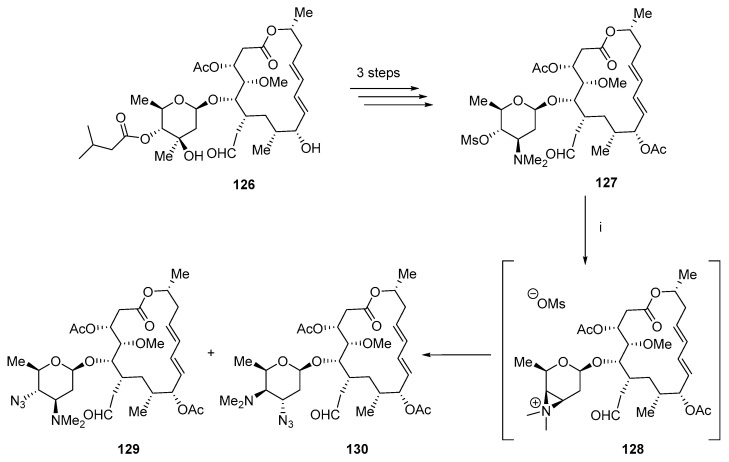
Synthesis of josamycin derivatives **129** and **130**. Conditions: (i) NaN_3_, DMF, 80 °C, 35% for **129**, 53% for **130**.

Similar results were reported to synthesize unsaturated josamycin derivatives (Scheme 33) [75]. The key mesylated intermediate **127** was prepared in three steps from josamycin **126**. Sodium azide reacts with **127** through azirium salt **128** to produce a mixture of isomers **129** and **130**.

### 3.2. Cis-Configuration

**Figure 7:**
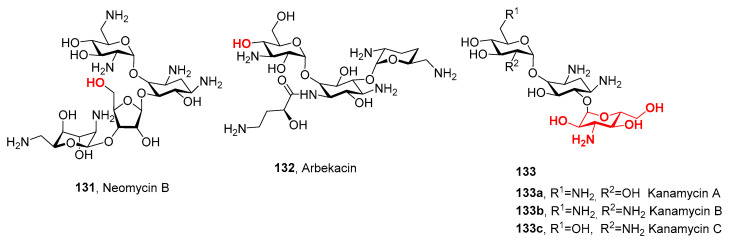
Structures of Neomycin B, Arbekacin, and Kanamycin.

Glycoside antibiotics were derivatized by introducing nitrogen functionalities in the *cis*-configuration. Arbekacin, kanamycin B, and neomycin B are the most important antibiotics. For example, Arbekacin strongly inhibits methicillin resistant *Staphylococcus aureus* [76], kanamycin shows activity against the gram-negative bacteria *E. coli* and *Klebsiella pneumonia* [77], and neomycin B can be used to cure liver encephalopathy [78]. Subsequent derivatization of these glycoside antibiotics is highlighted in red (Figure 7).

**Scheme 34:**
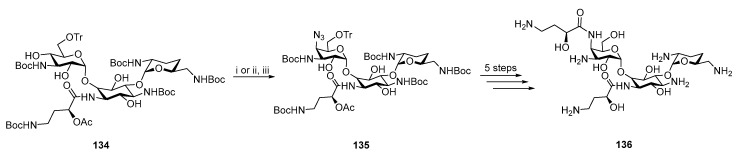
Activation and subsequent substitution in Arbekacin. Conditions: (i) MsCl, pyridine, (ii) Tf_2_O, and (iii) NaN_3_, 74% (two steps).

Sasaki et al. activated the hydroxyl group of Arbekacin by mesylation and subsequent substitution with sodium azide to obtain **135**, which was further converted in five steps to the final product **136** (Scheme 34) [76]. **136** has lower biological activity than Arbekacin **132**. This paper confirmed the results of Hiariwa et al., where substituting the hydroxyl group with a nitrogen-containing functional group resulted in a derivative with decreased biological activity [79].

**Scheme 35:**
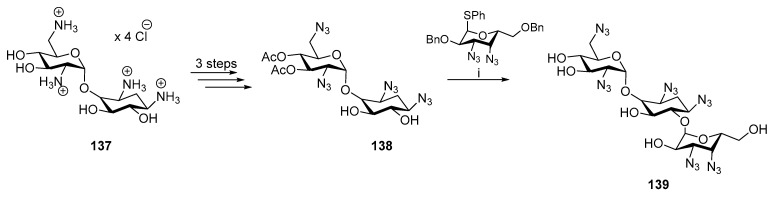
Glycosylation using an anomeric thioether group to produce Kanamycin B derivative **139**. Conditions: (i) NIS, TfOH, Et_2_O/DCM, and then, MeONa, MeOH/THF, 45%.

An alternative strategy using *O*-glycosylation can be used for the aza analogue kanamycin B (Scheme 35). The *cis*- configuration was introduced by *O*-glycosylation with 3,4-dinitrogen-containing carbohydrate. However, **139** exhibited lower minimum inhibitory concentration (MIC) than the original Kanamycin B [80].

**Scheme 36:**
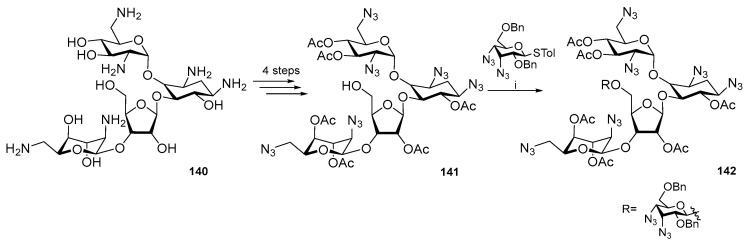
Glycosylation using the anomeric thioether group to produce neomycin B derivative **142**. Conditions: (i) NIS, TfOH, DCM, 81%.

A similar approach was used to synthesize neomycin B derivatives, where tolyl was used instead of phenyl in the anomeric thioether group (Scheme 36) [81,82]. The furanose hydroxyl group was derivatized. Biological testing was performed after the azide reduction and deacetylation to show lower activity against bacterial strains.

### 3.3. Methods Resulting in Cis- and Trans-Configurations

**Scheme 37:**
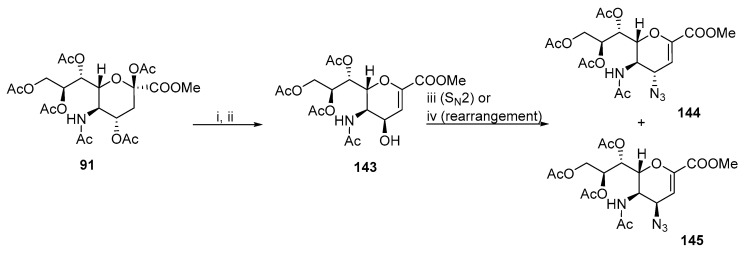
Introduction of the azide functionality into the Neu5Ac molecule. Conditions: (i) CF_3_SO_3_Si(CH_3_)_3_, ACN, 82%; (ii) TFA, ACN/water, 77%; (iii) HN_3_, TPP, DEAD, toluene, 67% for **144**, 34% for **145**; and (iv) HN_3_, TPP, DEAD, THF 17% for **144**, 52% for **145**.

Some methods provide a mixture of *trans*- and *cis*-stereoisomers with nitrogen functionalities at positions 3 and 4. The formation of a major product usually depends on the reaction conditions. Zbiral and coworkers incorporated the azide functionality into the Neu5Ac molecule [83,84,85]. Substituting the hydroxyl group with an azide in **143** under Mitsunobu conditions produces two isomers **144** and **145** (Scheme 37). The ratio of isomers depends on the solvent. Toluene facilitates the S_N_2 reaction and predominantly produces **144** (ratio of **144:145** = 3:1), whereas THF favors the 3,3-rearrangement and produces **145** (ratio of **144:145** = 2:3) as the major product. Both **144** and **145** were subsequently reduced by the Staudinger protocol to the corresponding amines.

**Scheme 38:**
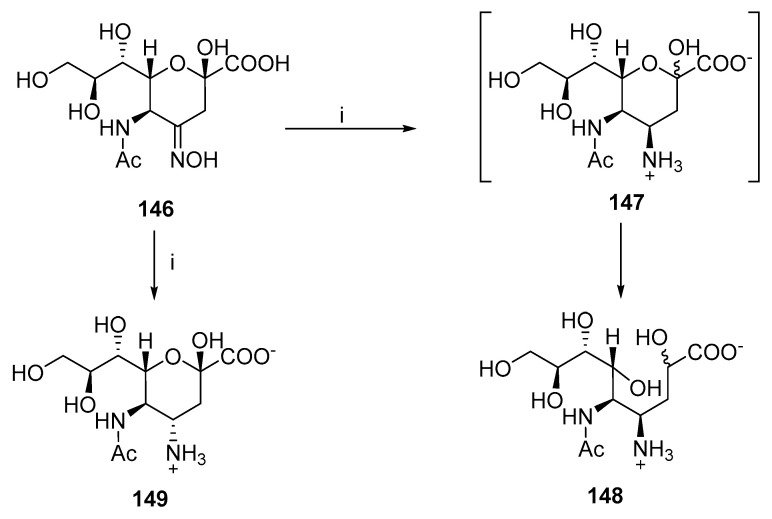
Reduction of the oxime functionality. Conditions: (i) H_2_, Pd/C, HCl, MeOH, 85%.

Another example is the reduction of the oxime functionality (Scheme 38) [86]. The catalytic hydrogenation of **146** produces two isomers: **147** and **149**. Stereoisomer **147** is unstable due to *syn-diaxial* interactions, which immediately undergoes a ring-opening reaction and subsequently reduces the keto group to afford **148**.

## 4. Nitrogen Functionalities at Positions 2, 3, and 4

**Scheme 39:**
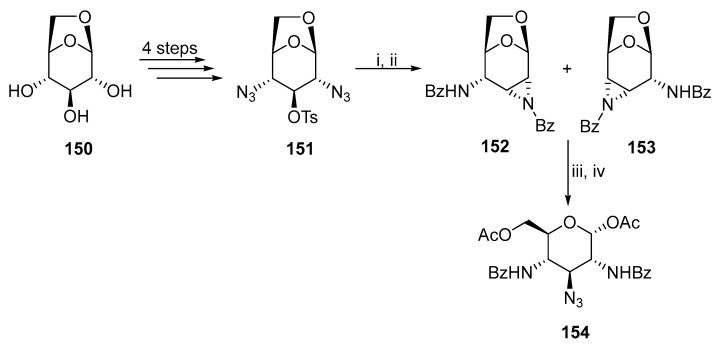
Synthesis of hexopyranoside **154** with three nitrogen functionalities using the aziridine ring-opening reaction. Conditions: (i) H_2_, Pd/C, HCl, EtOAc/EtOH; (ii) Bz_2_O, DMAP, pyridine/DCM, 83% (2 steps); (iii) LiN_3_, Al_2_O_3_, DMF/toluene; and (iv) TFA, Ac_2_O, 79% (two steps).

The study of glycoside antibiotics containing polyaminated pyranoses motivated the development to synthesize 2,3,4-tri- or 2,3,4,6-tetra-substituted D-hexopyranosides with nitrogen functionalities. Derivatives of D-hexopyranosides containing nitrogen functionality at positions 2, 3, and 4 are rare in comparison to disubstituted derivatives. The first synthesis is based on the aziridine ring-opening reaction. Bailliez and coworkers published a synthesis (Scheme 39), where levoglucosan **150** was converted to diazide **151** [87]. Subsequent reduction and benzoylation produced a mixture of isomers **152** and **153**. The mixture was treated with lithium azide and TFA to produce hexopyranoside **154** with three nitrogen functionalities without further purification.

**Scheme 40:**
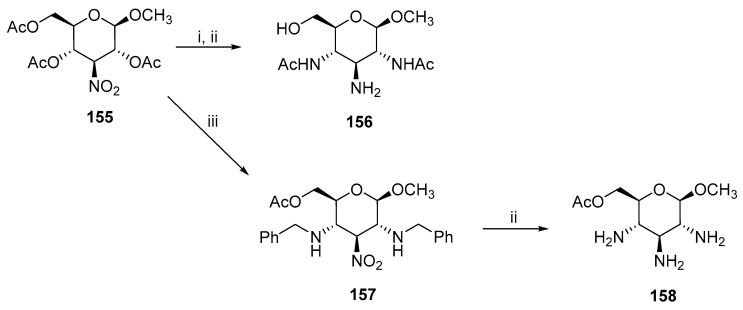
Sequential acetoxy group elimination to synthesize D-glucopyranosides with nitrogen functionalities at positions 2, 3, and 4. Conditions: (i) NH_3_/MeOH, then Ac_2_O, 57%; (ii) H_2_, Pd/C, MeOH, (87–90%); and (iii) BnNH_2_, CHCl_3_, 35%.

Another synthesis used sequential acetoxy group elimination with a subsequent Michael addition with ammonia or benzylamine (Scheme 40) [88,89,90]. The substitution at C2 and C4 carbons provided **156** and **157**, which was transformed into the corresponding triamine derivative **158**.

**Scheme 41:**
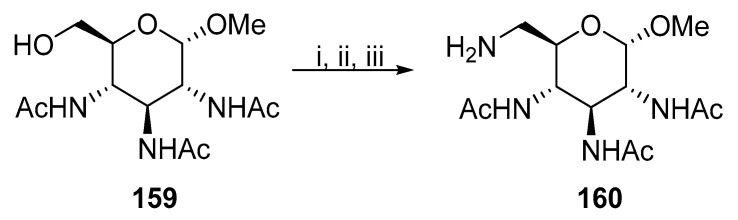
Synthesis of 2,3,4,6-tetraaminoglucopyranosid. Conditions: (i) MsCl, pyridine, 56%; (ii) NaN_3_, water, (51%); and (iii) H_2_, PtO_2_, HCl, water, 67%.

Tetra-substituted hexopyranosides with nitrogen functionalities were synthesized via trisubstituted precursors. The C-N bond at position 6 was introduced in the last step or simultaneously through substitution at other positions. The first procedure was published by Baer and coworkers [91]. The primary hydroxyl group of **159** was mesylated and substituted with the azide anion, and the azide was subsequently reduced to give **160** (Scheme 41). A similar protocol was used by Cleophax [87] and Meyer zu Reckendorf [92]. 

**Scheme 42:**
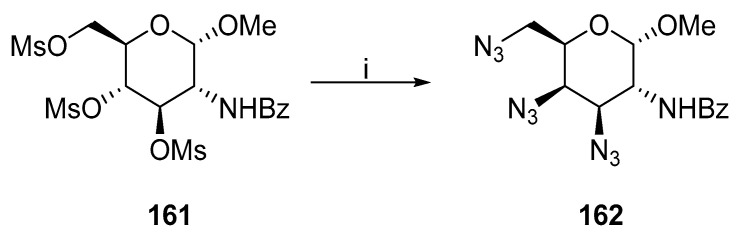
Synthesis of galactopyranoside with nitrogen functionalities at positions 2, 3, 4, and 6. Conditions: (i) NaN_3_, hexamethylphosphoric triamide, water, (10–15%).

Ali and coworkers synthesized **162** from glucopyranoside **161**, where the *galacto*-configuration arose from the *in-situ* formed oxazoline ring and its ring-opening reaction with the azide anion and a simultaneous two-fold mesylate substitution (Scheme 42) [93]. A similar procedure was used for galactopyranoside or idopyranoside [94,95].

## 5. Conclusions

The nitrogen functionalities of D-hexopyranosides increase their structural diversity, which results in numerous derivatives with interesting biological activities. With ongoing basic research on the use of these compounds for advanced biological studies and the identification of other nitrogen-containing hexopyranosides with improved biological effects and selectivity, synthetic methods that introduce various nitrogen functionalities were developed. The first methods to synthesize derivatives with vicinal nitrogen-containing functionalities emerged in the 1960s. These methods use the ring-opening reactions of aziridine and oxazoline intermediates. In addition to conventional substitution reactions of tosylated or mesylated hydroxyl groups, the Michael addition reaction is frequently studied with nitrogen nucleophiles to produce Michael adducts from nitro-olefins. Other methods such as oxime reduction and ring-opening reactions of imidazoline derivatives further supplement the synthetic methodology. Moreover, the developed synthetic methods introduce nitrogen functionalities in various oriented configurations. Finally, considering the substantial expansion of photoredox catalysis, we can expect the development of novel methods using highly reactive radical intermediates.

## Data Availability

Not applicable.
